# Prognostic analysis of extrameningeal solitary fibrous tumor using the modified Demicco model: a clinicopathologic study of 111 Chinese cases

**DOI:** 10.3389/fonc.2023.1272090

**Published:** 2024-01-04

**Authors:** Chen-chen Yao, Jian Zhou, Xiao Li, Jun Yang, Gang Chen, Jia Wei, Qin-he Fan, Qi-xing Gong

**Affiliations:** ^1^ Department of Pathology, The First Affiliated Hospital of Nanjing Medical University, Nanjing, China; ^2^ Department of Pathology, Women’s and Children’s Hospital Affiliated to Xiamen University (Xiamen Maternal and Child Health Care Hospital), Xiamen, China; ^3^ National Health Commission(NHC) Contraceptives Adverse Reaction Surveillance Center, Nanjing, China; ^4^ Jiangsu Provincial Medical Key Laboratory of Fertility Protection and Health Technology Assessment, Nanjing, China; ^5^ Jiangsu Health Development Research Center, Nanjing, China; ^6^ Department of Pathology, Affiliated Nanjing Drum Tower Hospital of Nanjing University School of Medicine, Nanjing, China

**Keywords:** solitary fibrous tumor, prognosis, risk model, TP53, TERT

## Abstract

**Introduction:**

Solitary fibrous tumor (SFT) represents a fibroblastic neoplasm exhibiting *NAB2::STAT6* gene rearrangement, displaying diverse clinical manifestations, spanning from benign to malignant. To predict prognosis, the modified (four-variable) Demicco (mDemicco) model was introduced. This investigation aims to authenticate the mDemicco risk model’s precision in Asian patients while investigating the clinicopathological and molecular factors linked to the prognosis of extrameningeal SFTs.

**Methods:**

Clinicopathological data from 111 extrameningeal SFT cases in East China, covering the period from 2010 to 2020, were thoroughly analyzed. The tumors were classified using the mDemicco model. Immunohistochemical evaluation of P16 and P53, molecular detection of *TP53* and *TERT* promoter mutation, and fluorescence *in situ* hybridization for *CDKN2A* gene alterations were performed. Statistical methods were utilized to assess the associations between clinicopathological or molecular factors and prognosis.

**Results:**

Histologically, only one parameter, the mitotic count, exhibited a statistical correlation with progression-free survival (PFS) and overall survival (OS). During the Kaplan-Meier analysis, the variation in PFS among the different risk groups exhibited a notable trend towards statistical significance. Nevertheless, 3 out of 74 patients classified as low-risk SFTs and 7 out of 21 patients classified as intermediate-risk exhibited disease progression. Among the 5 patients with *TP53* mutations and/or mutant-type P53 immunophenotype, 3 experienced disease progression, including 2 intermediate-risk patients. Additionally, among the 4 patients with *TERT* promoter mutations who were followed up, 3 showed progression, including 2 intermediate-risk patients. Moreover, it was observed that hemizygous loss of *CDKN2A* was detected in more than 30% of one case, yet the patient exhibited a favorable survival outcome.

**Conclusion:**

The mDemicco risk model exhibits certain limitations when dealing with smaller tumor sizes, younger age groups, and occurrences of malignant and dedifferentiated SFTs. Furthermore, molecular factors, such as *TP53* or *TERT* promoter mutations, may identify intermediate-risk SFTs with poorer prognoses.

## Introduction

Solitary fibrous tumor (SFT) is a fibroblastic neoplasm characterized by *NAB2::STAT6* gene fusions and can occur in various anatomical sites, displaying a wide morphological spectrum. Formerly considered an intermediate tumor with some incidence of metastasis or recurrence (1), the clinical behavior of individual SFTs remains challenging to predict. Notably, even morphologically classical SFTs may exhibit malignant biological behavior. Meningeal SFTs have a higher propensity for metastasis compared to extrameningeal SFTs (2). For central nervous system (CNS) SFTs, the WHO CNS classification categorizes them as grade 1, 2, or 3 based on clinicopathological variables, including mitotic figures and necrosis (3). However, for extrameningeal SFTs, the Demicco metastatic risk model is recommended for evaluating metastatic risk, as outlined in the fifth edition of the WHO Classification of Soft Tissue and Bone Tumors. This model integrates mitotic count (≥2 mitoses/mm^2^), patient age (≥55 years), and tumor size stratified into 5 cm tiers, classifying tumors into low, intermediate, and high-risk groups (4). Subsequently, the mDemicco model was refined and validated to include necrosis (≥10%) as a fourth variable (5). In a comparative study by Demicco et al., the mDemicco model was assessed alongside the Pasquali and Salas models for predictive efficacy and accuracy. The findings indicated that the mDemicco model and Salas model were the most reliable in predicting metastasis, while the mDemicco model demonstrated higher accuracy in risk assessments (6). Notably, Machado et al. proposed that molecular genetic factors may serve as new predictive factors in SFTs (7). However, it is noteworthy that the variables considered in the aforementioned models predominantly involve morphological and clinical features, with limited inclusion of immunohistochemical and molecular features. In our study, we explore clinical, morphological, immunohistochemical, and molecular features associated with prognosis to provide more accurate predictions of the biological behavior of SFTs.

## Materials and methods

### Patients and samples

A total of 111 cases of primary extrameningeal SFTs were identified from the Department of Pathology at the First Affiliated Hospital of Nanjing Medical University from 2010 to 2020. The diagnosis of SFT in our cohort was based on histological features, further supported by ancillary immunohistochemical tests. Cases with atypical morphology or abnormal immunophenotype were confirmed to harbor *NAB2::STAT6* fusion genes using next-generation sequencing (NGS) tests. Inclusion criteria encompassed patients who underwent complete surgical resection with negative margin. Exclusion criteria involved cases with incomplete records, insufficient tumor sections for experimentation, or deaths due to unrelated causes. Clinical data (age, gender, tumor site, size, treatment, chemotherapy/radiotherapy) and follow-up data (recurrence, metastases, and final outcome) were collected. Formalin-fixed, paraffin-embedded tissue (FFPET) was obtained from the corresponding files.

### Histopathology

All available H&E slides were independently examined by at least two experienced soft tissue pathologists. Data retrieved included mitotic count, assessed in the most mitotically active areas per 10 high-power fields (1 HPF=0.2289mm^2^), and categorized as 0/10HPF, 1-3/10HPF, or ≥4/10HPF. Additional parameters assessed were necrosis (<10%, ≥10%), cellularity (low, moderate, high), tumor invasion (yes, no), boundary (capsule, no-capsule, infiltration), and morphological subtypes. Evaluation of the above histopathological variables was carried out following a prior study by Demicco et al. (6). All cases were stratified into low, intermediate, and high-risk groups using the mDemicco risk model (**Table 1**).

**Table 1:** Modified four-variable risk stratification model for development of metastasis in solitary fibrous tumors.

Risk factor	Score
*Age*	
<55	0
≥55	1
*Tumor size(cm)*	
<5	0
5 to<10	1
10 to<15	2
≥15	3
Mitotic count (/10HPF)	
0	0
1-3	1
≥4	2
*Tumor necrosis*	
<10%	0
≥10%	1
*Risk class*	*Total score*
Low	0-3
Intermediate	4-5
High	6-7

### Tissue microarray, immunohistochemistry, FISH

Two tissue microarrays (TMA) were constructed from 53 SFT pathology blocks, using 2mm core size to obtain the most representative areas. Immunohistochemical (IHC) staining was conducted on 3µm thick sections of FFPET from both TMAs. Antibodies against STAT6 (MXR031, RMA-1066), P53 (MX008, MAB-0674), and P16 (MX007, MAB-0673) were purchased from Fuzhou Maixin Biotect Co., Ltd. The Envision system (Dako, Glostrup, Denmark) was utilized to detect the reactions. Nuclear expression of P53 was classified into two types: over 50% strong positive expression and complete negative expression were considered as mutant-type P53 expression, while less than 50% positive or weak expression was considered as wild-type P53 expression (8). Expression of P16 was categorized into two groups based on the percentage of positive cells in the total tumor cell population: more than 5% or less than 5% (7). For fluorescence *in situ* hybridization (FISH) assays, commercial *CDKN2A* probes (Anbiping, Guangzhou, China) were used on 3 µm thick sections from FFPET blocks to detect gene deletions. A minimum of 50 tumor nuclei were scored, and the cut-off value was established based on the proportion of cells carrying the abnormal pattern: 30% for homozygous and hemizygous deletions, and 40% for monosomy.

### TP53, TERT promotion mutation analyses

DNA isolation from 53 paraffin samples was performed using the QIAamp DNA FFPE Tissue Kit (Shanghai Yuanqi Biomedical Technology Co. Ltd., China). Telomerase reverse transcriptase (*TERT*) was detected using the amplification refractory mutation system (ARMS) PCR method to identify the two most common *TERT* promoter mutations C250T and C228T, employing the AmoyDx@ *TERT/HRAS* Mutations Detection Kit. For *TP53* mutations (exon 5-8) examination, sanger sequencing was conducted using the *TP53* Mutations Detection Kit from Shanghai Yuanqi Biomedical Technology Co. Ltd., China, following the manufacturer’s instructions.

### Statistical analyses

Patient outcomes were analyzed using Kaplan-Meier analysis. The log-rank test determined the prognostic significance of individual risk factors, including mDemicco stratification, site, age, gender, tumor size, mitotic count, necrosis, cellularity, tumor invasion, boundary, P53 staining, P16 staining, *TP53* mutations, and *TERT* promoter mutations. Multivariable Cox regression analyses were not performed due to most data not fitting the proportional hazards assumption. The chi-squared test was employed to assess the association of *TP53* mutations or *TERT* promoter mutations with site, age, gender, tumor size, mitotic count, necrosis, cellularity, tumor invasion, boundary, P53 staining, and P16 staining. Chi-square tests were also used to analyze the significance of differences between risk groups in *TP53* mutations, *TERT* promoter mutations, and P16 staining. For visualize *TP53/TERT* alterations, we used Oncoprint plot by R package. Progression-free survival (PFS) was defined as the time from tumor excision until death, metastasis, or relapse, with surviving progression-free patients censored at the date of last follow-up. Overall survival (OS) was defined as the time observed from tumor excision until death, with surviving patients censored at the date of last follow-up date. Analyses were performed using SPSS software version 25.0 for Windows, and statistical significance was considered for an alpha of P<0.05.

## Results

### Clinical features

The study included a total of 111 patients, consisting of 42 men and 69 women. The median age at diagnosis was 54 years (range 19 to 82 years). Tumors were most commonly located in the intrapleural region (62 cases, 55.6%), followed by the abdomen/pelvis (29 cases, 26.1%), head and neck (12 cases, 10.8%), trunk (4 cases, 3.6%), and extremities (4 cases, 3.6%). All patients underwent surgical resection with curative intent, with 3 patients receiving preoperative therapy through embolization, and an additional 3 patients receiving postoperative radiotherapy. Clinical follow-up data were available for 97 patients (87.4%), and the median clinical follow-up time was 53 months (range 1 to 136 months). 10 patients experienced local recurrence after resection, 4 patients developed metastatic disease, and 3 patients succumbed. Further details of the clinical information and follow-up data for progressive SFT cases are presented in **Table 2**. The median time to the first recurrence was 52 months (range 6 to 108 months), while the median time to the first metastasis was 30.5 months (range 8 to 97 months). Overall, the 5-year and 10-year progression-free rates were 91.8% and 87.6%, respectively, and the survival rates were 96.9% for both 5-year and 10-year intervals.

**Table 2:** Clinical information and follow-up data of progressive SFT cases.

No	Gender	Age	Site	Size(cm) (Maximum diameter)	Mitotic(/10HPF)	Necrosis score	Histologic subtype	mDemicco risk stratification	*TP53* mutation	*TERT* promoter mutation	Treatment	Recurrence and/or metastasis	Sites of recurrence and metastasis	PFS(M)	Outcome
1	Female	60	Nasal	3.0	≥4	0	Classical	Low	WT	WT	Resection+ radiotherapy	Recurrence	Nasal	50	Alive
2	Female	65	Nasal	1.5	1-3	0	Classical	Low	WT	WT	Resection	Recurrence	Nasal	6	Alive
3	Female	43	Kidney	4.5	1-3	0	Malignant	Low	WT	WT	Resection+ radiotherapy	Recurrence	Kidney	54	Alive
4	Man	30	Abdomen	9.2	≥4	0	Classical	Intermediate	WT	MUT	Resection	Recurrence and metastasis	Liver	26	Death
5	Female	57	Intrapleural	2.5	≥4	0	Malignant	Intermediate	MUT	WT	Resection	/	/	41	Death
6	Female	45	Intrapleural	15	1-3	0	Classical	Intermediate	WT	WT	Resection	Recurrence	Intrapleural	108	Alive
7	Female	58	Intrapleural	5.5	≥4	0	Classical	Intermediate	WT	MUT	Resection	Recurrence	Intrapleural, involved breast	73	N/A
8	Female	63	Intrapleural	14	1-3	0	Classical	Intermediate	WT	WT	Resection	Recurrence	Intrapleural	79	Alive
9	Female	52	pelvic	12.5	≥4	0	Classical	Intermediate	–	–	Resection	Recurrence	Pelvic	57	Alive
10	Female	69	pelvic	10.5	≥4	0	Malignant	Intermediate	WT	WT	Resection	Recurrence and metastasis	Abdomen and pelvic	8	N/A
11	Female	60	abdomen	20	≥4	0	Classical	High	MUT	MUT	Resection+ endocrine therapy+ radiofrequency ablation	Recurrence and metastasis	Multiple	35	Death
12	Man	64	Retroperito-neal	15	≥4	1	Malignant	High	WT	WT	Resection+ targeted therapy	Metastasis	Multiple	97	Alive

SFT, solitary fibrous tumor; WT, wild-type; MUT, mutation; N/A, not available; “-”, not detected.

"/", no recurrence or metastasis.

### Pathological features

**Figure 1:**
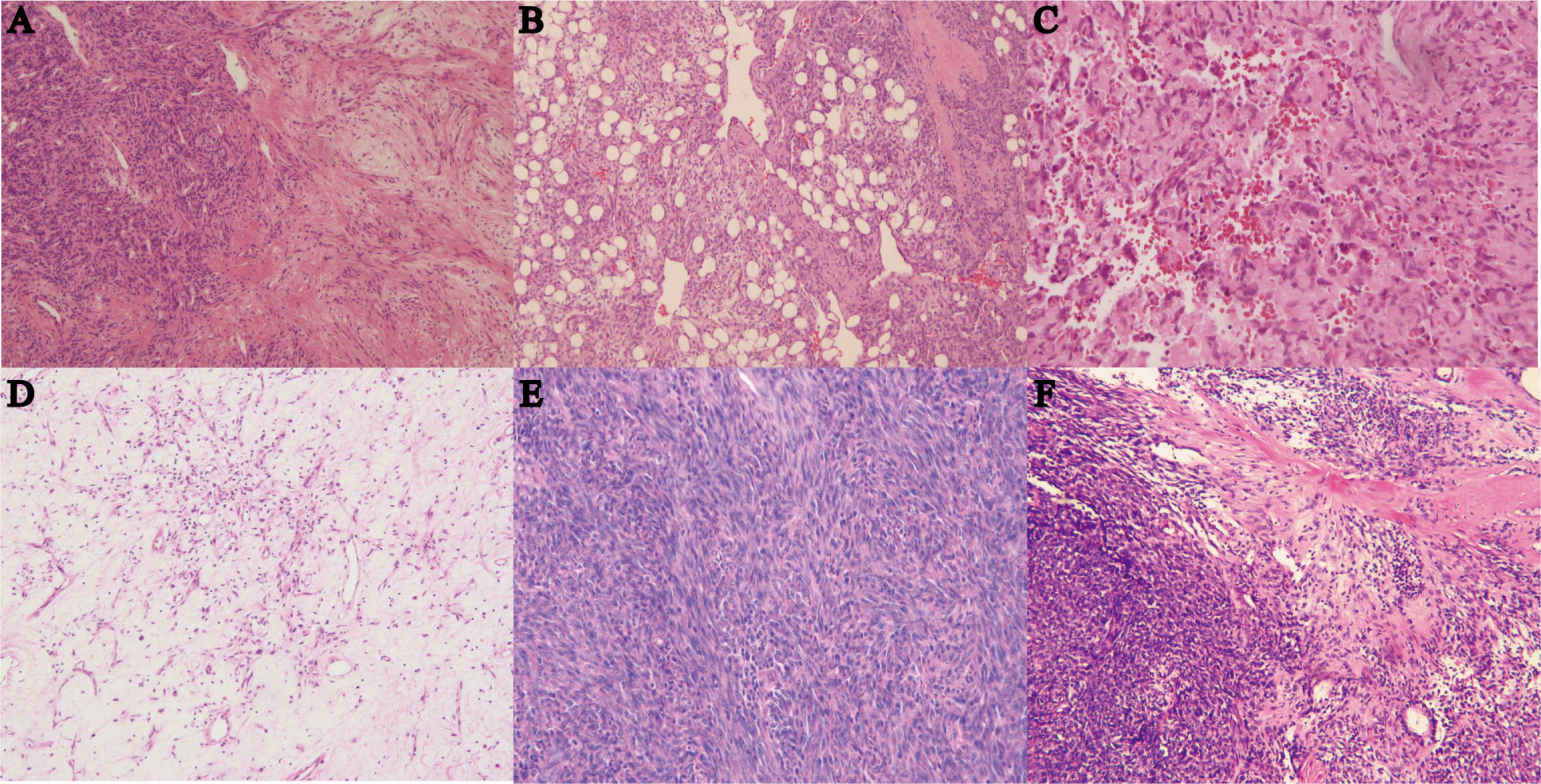
**(A)** Hematoxylin and eosin (HE) staining of classical SFT with gentle spindle cells and abundant collagen, magnification ×100. **(B)** HE staining of fat-forming SFT with a component of mature adipose tissue, magnification ×100. **(C)** HE staining of giant cell-rich SFT with a population of multinucleated giant cells in conventional SFT, magnification ×100. **(D)** HE staining of myxoid SFT with more than 50% myxoid change area, magnification ×100. **(E)** HE staining of malignant SFT showing cellularity, morphology atypia, active mitotic figures, and probably necrosis, magnification ×100. **(F)** HE staining of dedifferentiated SFT showing high-grade sarcoma with classical SFT component or history, magnification ×40.

Tumor sizes ranged from 1 to 24 cm (median 6 cm). Tumor necrosis was present in ≥10% of cases in 10 instances. Mitotic figures were observed rarely in most tumors, with 36 cases showing no mitosis, 53 cases displaying 1 to 3 mitoses per 10 high-power fields (10HPF), and 22 cases exhibiting more than 4 mitoses per 10HPF. Most tumors were at least moderately cellular, with 34 cases demonstrating low cellularity and 33 cases showing high cellularity. In terms of the boundary, 33 cases exhibited an infiltrative pattern. All cases were classified into different subtypes: classical SFT (88 cases, 79.3%) (**Figure 1A**), fat-forming SFT (2 cases, 1.8%) (**Figure 1B**), giant cell-rich SFT (3 cases, 2.7%) (**Figure 1C**), myxoid SFT (8 cases, 7.2%) (**Figure 1D**), malignant SFT (6 cases, 5.4%) (**Figure 1E**), and dedifferentiated SFT (4 cases, 3.6%) (**Figure 1F**).

### Validation of the mDemicco metastic risk model

**Figure 2:**
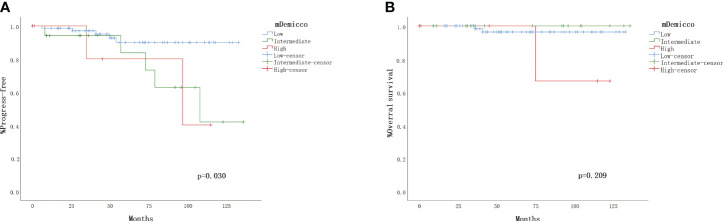
Validation of the four-variable risk stratification score. **(A)** Kaplan–Meier plot for time to progress by risk group. **(B)** Kaplan– Meier plot for overall survival.

All cases were categorized into different risk groups based on the mDemicco risk model, with 79 cases (71.4%) classified as low-risk, 23 cases (20.5%) as intermediate-risk, and 9 cases (8.0%) as high-risk. Clinical follow-up data were available for 74 (76.3%) of the low-risk cases, 17 (17.5%) of the intermediate-risk cases, and 6 (6.2%) of the high-risk cases, as presented in **Table 3**. In the Kaplan-Meier analysis (**Figures 2A, B**), the difference in PFS among the risk groups showed a trend towards statistical significance (log-rank P=0.030). Specifically, the PFS rate of low-risk SFTs was higher compared to intermediate-risk and high-risk SFTs, resulting in better outcomes. However, it is noteworthy that some patients in the low-risk group experienced disease progression. The Kaplan-Meier curve of the low-risk group tended to separate from that of the intermediate-risk and high-risk groups, while the PFS rate and Kaplan-Meier curves had little difference between the intermediate and high-risk groups. Conversely, the correlation between OS rate and mDemicco risk groups did not reach statistical significance (log-rank P=0.209). Initially, there was little difference in OS and Kaplan-Meier curves among the groups. However, with a longer follow-up period, the Kaplan-Meier curves began to show a trend of separation.

**Table 3:** Risk class sizes and relative outcomes of modified risk stratification models.

mDemicco		PFS		OS	
	N (%)	-5 years (%)	-10 years (%)	-5 years (%)	-10 years (%)
Low	74 (76.3)	95.9% (71)	95.9% (71)	100% (74)	100% (74)
Intermediate	17 (17.5)	76.5% (13)	58.8% (10)	88.2% (15)	88.2% (15)
High	6 (6.2)	83.3% (5)	66.7% (4)	83.3% (5)	83.3% (5)
	97	91.8% (89)	87.6% (85)	96.9% (94)	96.9% (94)

### PFS and OS of TMA samples

**Figure 3:**
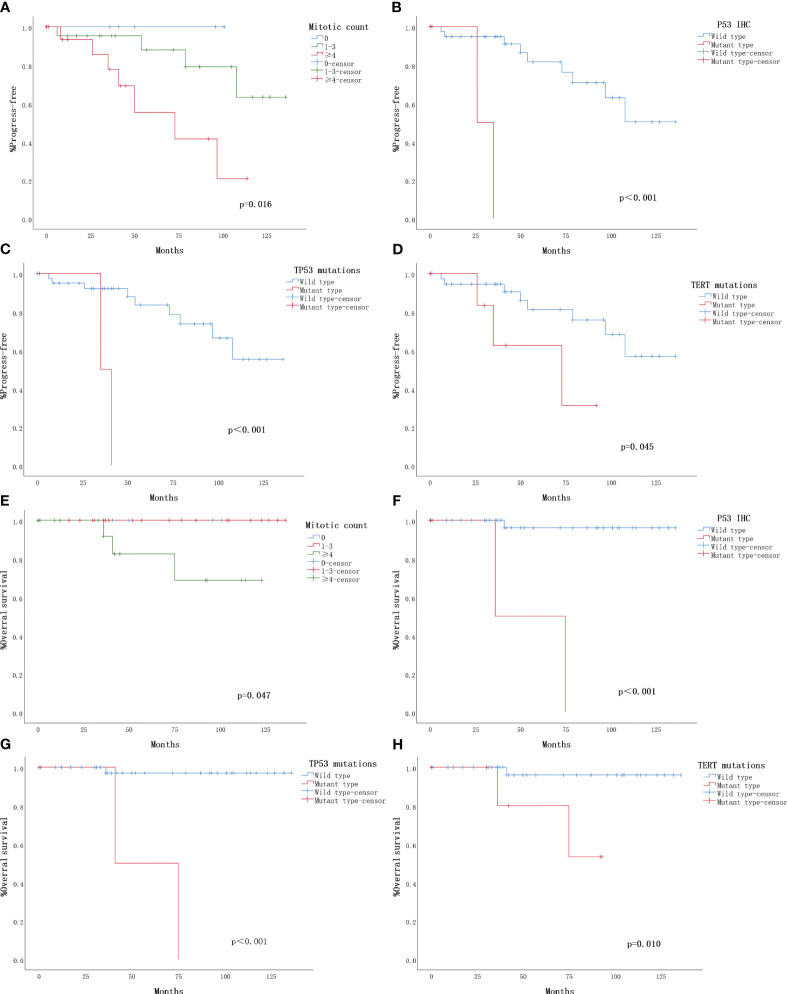
Clinical outcomes for 53 solitary fibrous tumors with follow-up. The Kaplan-Meier plots depict overall survival and progression-free survival for the 53 solitary fibrous tumors (SFTs) with follow-up. **(A)** Kaplan-Meier plot illustrating the time to progression based on mitotic count. **(B)** Kaplan-Meier plot showing the progression based on P53 immunohistochemistry (IHC). **(C)** Kaplan-Meier plot indicating the progression based on TP53 mutation. **(D)** Kaplan-Meier plot demonstrating the progression based on TERT promoter mutation. **(E)** Kaplan-Meier plot displaying overall survival based on mitotic count. **(F)** Kaplan-Meier plot depicting overall survival based on P53 immunohistochemistry (IHC). **(G)** Kaplan-Meier plot illustrating overall survival based on TP53 mutation. **(H)** Kaplan-Meier plot showing overall survival based on TERT promoter mutation.

The TMA sample consisted of 24 (45.3%) cases classified as low-risk, 21 (39.6%) cases as intermediate-risk, and 8 (15.1%) cases as high-risk. Survival curve analysis (Kaplan-Meier curve) was performed for each clinicopathological finding, and the statistical data are presented in **Table 4**. Representative survival curves are illustrated in **Figures 3A-D**. Factors influencing the prognosis of SFTs progression included mitotic count (log-rank P=0.016), P53 immunohistochemistry (log-rank P<0.001), *TP53* mutation (log-rank P<0.001), and *TERT* promoter mutation (log-rank P=0.045). No significant differences were observed in disease progression based on sex, age, site, tumor size, necrosis, cellularity, tumor invasion, boundary, or P16 staining. Regarding overall survival, mitotic count (log-rank P=0.047), P53 immunohistochemistry (log-rank P<0.001), *TP53* mutation (log-rank P<0.001), and *TERT* promoter mutation (log-rank P=0.010) demonstrated statistically significant correlations (**Figures 3E-H**). However, no significant associations were found between overall survival and age, sex, site, tumor size, necrosis, cellularity, tumor invasion, boundary, or P16 staining.

**Table 4:** The correlation between the variables and PFS or OS.

Variables	PFS (log-rank P)	OS(log-rank P)
Age	0.260	0.767
Sex	0.704	0.640
Site	0.106	0.458
Size	0.864	0.832
Mitotic count	0.016	0.047
Necrosis	0.632	0.535
Cellularity	0.089	0.277
Tumor invasion	0.762	0.946
Boundary	0.055	0.439
P16 IHC	0.246	0.321
P53 IHC	<0.001	<0.001
TP53 mutation	<0.001	<0.001
TERT promoter mutation	0.045	0.010

### TP53 mutation and TERT promoter mutation

**Figure 4:**
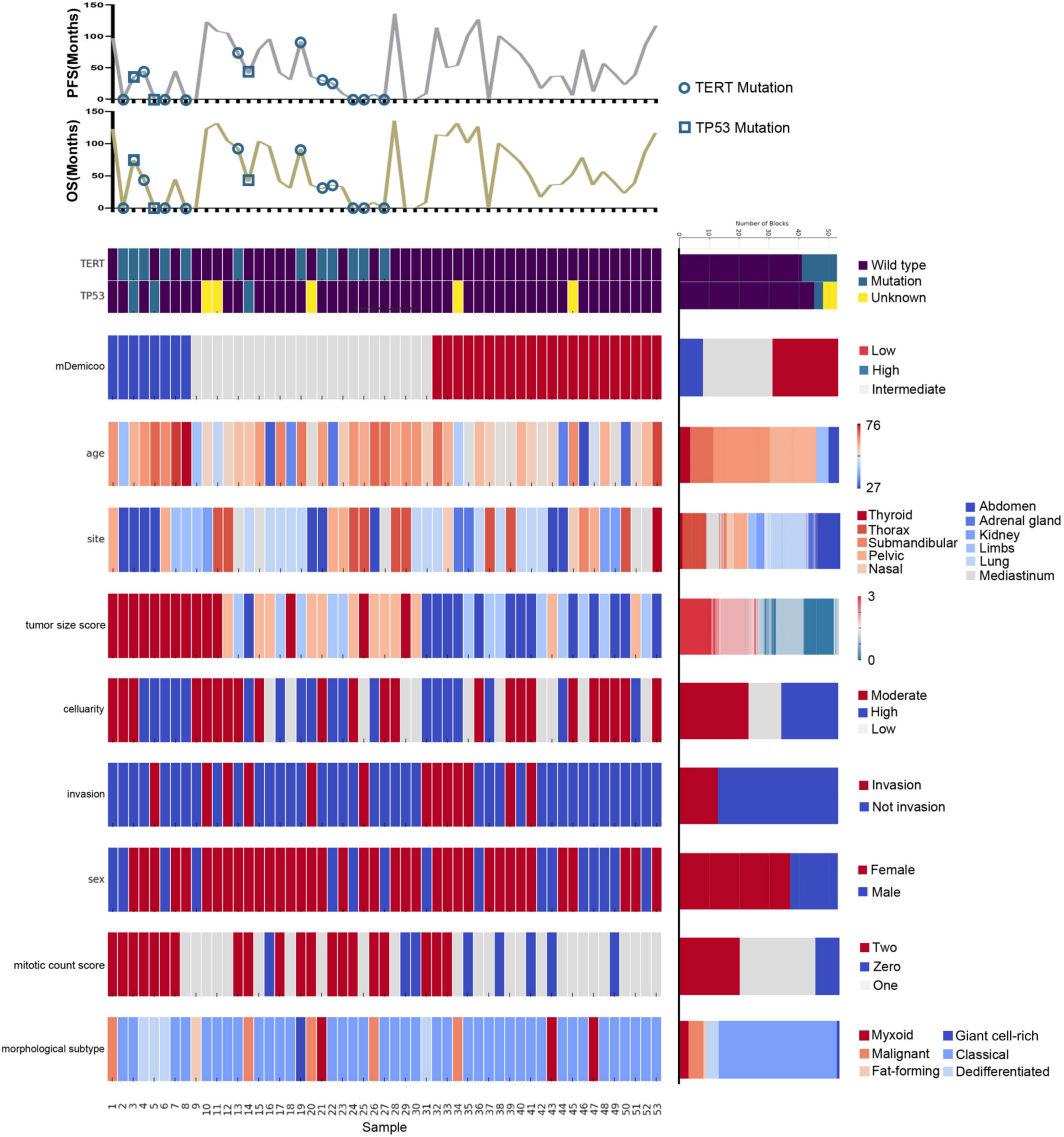
Landscape of *TERT/TP53* mutation status and pathological features of SFTs in TMAs. Oncoprint summarizing the enrichment of *TERT/TP53* mutation status by risk groups. Oncoprint also show overlapping/separation relationships between different groups and clinicopathological factors.

**Figure 5:**
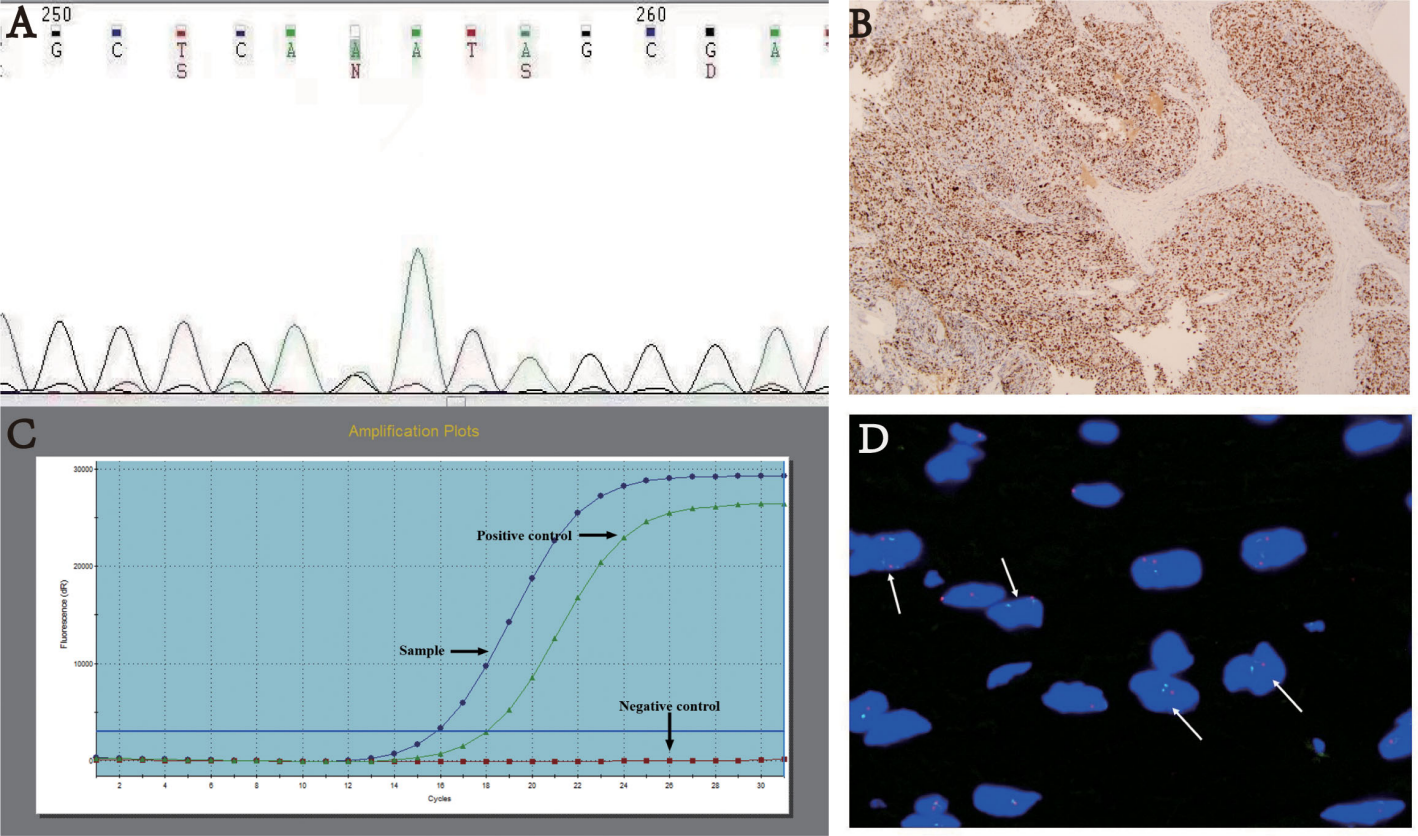
**(A)** TP53 mutation p. D184N. **(B)** Immunohistochemistry: Mutant-type P53 expression - strong and diffuse positive, magnification×40. **(C)** TERT promoter mutation. **(D)** FISH analysis of CDKN2A showing loss of heterozygosity with two green and one red signals.

The *TP53* mutation and *TERT* promoter mutation appeared to be concentrated in cases in the intermediate-risk group and high-risk group (**Figure 4**). The mutation rate of *TP53* was found to be 6.3% (3 out of 48 cases). All three cases with *TP53* mutation were located in exon 5, with one case showing P.D184N mutation, one case with P.D184G mutation (**Figure 5A**), and one case with P.C141G mutation. Two of these cases exhibited immunohistochemical P53 mutant-type expression (**Figure 5B**). Additionally, two cases showed mutant-type P53 expression, despite not detecting the *TP53* mutation in our study. This discrepancy could be attributed to potential issues such as unqualified DNA due to long-term preservation degradation or the methodological limitation of detecting only hotspot *TP53* gene mutations using Sanger sequencing. Among the five cases with *TP53* mutation or P53 mutant-type expression, three were classified as high-risk SFTs, and two were intermediate-risk SFTs. Among the four patients followed up, three (75%) experienced disease progression, including two intermediate-risk patients (**Table 5**).

**Table 5:** Prognostic and pathological features of SFTs with TP53 mutations or mutant-type P53 expression.

No	*TP53* gene	P53 IHC	*TERT* promotermutation	Histologic subtype	mDemicco risk	outcome
1	p. D184N	MUT	MUT	Classical	High	Recurrence, multiple metastases, death
2	p. C141G	MUT	WT	Differentiated	High	NED
3	p. D184G	WT	WT	Malignant	Intermediate	Death
4	–	MUT	MUT	Classical	Intermediate	Recurrence, multiple metastases, death
5	–	MUT	MUT	Differentiated	High	N/A

SFT, solitary fibrous tumor; WT, wild-type; MUT, mutation; “-”, not detected; NED, non-evidence of disease; N/A, not available.

The mutation rate of *TERT* promoter (**Figure 5C**) was found to be 22.6% (12 out of 53 cases), comprising 0% (0 out of 23) in the low-risk group, 31.8% (7 out of 22) in the intermediate-risk group, and 62.5% (5 out of 8) in the high-risk group. The mutation rate was significantly higher in the high-risk group compared to the other groups (P<0.001). One patient exhibited both *TP53* mutation and *TERT* promoter mutation, and this case was classified as high-risk. Among the four patients followed up, three (75%) experienced disease progression, including two intermediate-risk patients. Among the intermediate-risk SFTs, the progression rate was 30.8% for *TERT* wild-type SFTs and 66.7% for *TERT* mutant-type SFTs (**Table 6**).

**Table 6:** Prognostic and pathological features of SFTs with *TERT* promoter mutation.

No	*TERT* promoter mutation	*TP53* gene mutation	Histologic subtype	mDemicco risk	outcome
1	MUT	WT	Classical	Intermediate	Recurrence
2	MUT	WT	Giant rich-cell	Intermediate	N/A
3	MUT	WT	Myxoid	Intermediate	NED
4	MUT	WT	Classical	Intermediate	Recurrence, metastases, death
5	MUT	WT	Classical	Intermediate	N/A
6	MUT	WT	Classical	Intermediate	N/A
7	MUT	WT	Classical	Intermediate	N/A
8	MUT	WT	Classical	High	N/A
9	MUT	MUT	Classical	High	Recurrence, multiple metastases, death
10	MUT	WT	Differentiated	High	N/A
11	MUT	WT*	Differentiated	High	N/A
12	MUT	WT	Classical	High	N/A

SFT, solitary fibrous tumor; WT, wild-type; MUT, mutation; “-”, not detected; “*”, mutant-type P53 immunophenotype; NED, non-evidence of disease; N/A, not available.

Interestingly, among the 12 patients who developed progression, four cases had *TERT* promoter mutations or *TP53* mutations, with three cases being classified as intermediate-risk and one case as high-risk.

### P16 IHC and CDKN2A FISH

*CDKN2A* heterozygous deletions (>30%) in tumor cells were detected in only one case (**Figure 5D**). This case was located in the groin and classified as intermediate-risk SFT. The P16 expression was approximately 50% positive. Encouragingly, there was no evidence of disease progression during the 30 months of follow-up.

The expression rate of P16 was found to be 46% (23 out of 49 cases), including 45.5% (10 out of 22) in the low-risk group, 35.5% (7 out of 20) in the intermediate-risk group, and 71.4% (5 out of 7) in the high-risk group. Although the expression rate was higher in the high-risk group, the difference between groups was not statistically significant (P=0.307).

## Discussions

Demicco et al. conducted a comparison of various risk models in a multi-institutional SFT cohort, which included mDemicco, Pasquali, Salas OS, Salas MET, and Salas LR, designed for extracranial or pleural SFTs. The results revealed that mDemicco and Sala OS exhibited the best predictive efficacy for metastasis and recurrence. mDemicco outperformed other models in identifying both low-risk and high-risk SFTs. In our study, we also observed a better predictive effect of the mDemicco system on PFS (p=0.030), but no statistical correlation was found between OS and the mDemicco system (p=0.209). Notably, three patients classified as low-risk experienced disease progression. Unlike the mDemicco study, where the low-risk group had no metastatic or fatal cases, these three cases showed no observable necrosis or necrotic area less than 10%. Additionally, all cases had tumor sizes smaller than 5cm, and one was diagnosed under the age of 55, possibly contributing to their low-risk scores in the model. Therefore, we can conclude that the mDemicco risk model still has limitations in accurately predicting SFT stratification and prognosis. Furthermore, among the three low-risk cases that experienced progression, one was diagnosed as malignant SFT, and two were classical SFTs. Malignant SFTs are known to exhibit more aggressive biological behavior. Malignant SFTs are often classified as intermediate-risk or high-risk SFTs (**Table 7**). These findings suggest that the mDemicco risk model should not be applied to predict the behavior of malignant SFTs. Hassani et al. (9) also confirmed the limitations of the mDemicco risk model in prognosticating aggressive tumor behavior. In their study, some patients classified as low risk according to Demicco’s model experienced distant metastases. They particularly highlighted the aggressive behavior of focal dedifferentiated tumors. Additionally, Yui chi yamda et al. (10) established dedifferentiation as an independently significant prognostic factor in their analysis of overall survival curves. The authors further suggested that central nervous system involvement, hypoglycemia, and dedifferentiation in recurrent or metastatic cases may worsen the subsequent clinical course. Hence, we can conclude that the mDemicco risk model is not suitable for risk assessment in cases of malignant and dedifferentiated SFTs. Moreover, two cases of recurrent classic SFTs occurred in the nasal cavity. Similar to SFTs occurring in the orbit (11), SFTs in the nasal cavity with lower risk scores did not align with their biological behavior. This inconsistency could be attributed to the restricted site resulting in smaller tumor sizes. Additionally, routine marginal resection due to the tumor’s particular location might have led to underestimated positive margins. Consequently, it is advisable to use the mDemicco model cautiously for predicting progression in rare sites (**Supplementary Table 1**).

**Table 7:** Prognostic and pathological features of malignant and differentiated SFTs.

No	Histologic subtype	*TERT* promoter mutation	*TP53* gene mutation	mDemicco risk	outcome
1	Malignant	WT	–	Low	Recurrence
2	Malignant	WT	MUT	Intermediate	Death
3	Malignant	WT	–	Intermediate	NED
4	Malignant	WT	WT	Intermediate	Recurrence, metastases
5	Malignant	WT	WT	High	Recurrence, metastases
6	Malignant	–	–	High	NED
7	Differentiated	WT	WT	Intermediate	NED
8	Differentiated	MUT	WT	High	N/A
9	Differentiated	WT	MUT	High	NED
10	Differentiated	MUT	WT*	High	N/A

SFT, solitary fibrous tumor; WT, wild-type; MUT, mutation; “-”, not detected; “*”, mutant-type P53 immunophenotype; NED, non-evidence of disease; N/A, not available.

We have also observed lower PFS in patients with intermediate-risk SFT compared to those with high-risk SFT in our data. However, when comparing our study with that of Hassani et al. (9), it’s important to note that our study cohort included a higher percentage of low-risk SFTs (76.3% vs. 62.7%) and a lower percentage of intermediate-risk SFTs (17.5% vs. 25.5%) and high-risk SFTs (6.2% vs. 32.4%). This difference in composition ratios between the two cohorts could have influenced the observed results. Additionally, the short follow-up duration and radical treatment in high-risk patients in our study may also contribute to the relatively superior PFS, and some intermediate-risk SFT cases might have been underestimated and undertreated. One of our study purposes is to identify this part of the underestimated intermediate -risk cases and give them adequate treatment.

To date, no risk stratification system has incorporated molecular factors into its classification. However, a study has suggested that *TP53* mutations and *TERT* promoter mutations could serve as new prognostic indicators for risk stratification models in SFTs. Particularly for SFTs in the intermediate-risk group, *TP53* mutation and/or *TERT* promoter mutation were strongly correlated with tumor aggressive behaviors (7). In our TMA series, we detected *TP53* mutations and/or mutant-type P53 immunophenotype in 5 cases. Since there was a statistically significant correlation between *TP53* mutations and P53 immunophenotype (p=0.007), and both *TP53* mutations and P53 immunophenotype were associated with SFTs progression, we propose that all the 5 cases possess activation of the oncogene pathway involving *TP53* gene dysfunction. In a study by PARK et al., almost all SFTs with *TP53* mutations were classified as high-risk, and *TP53* mutations were significantly associated with increased nuclear atypia, active mitosis, and an elevated Ki-67 proliferation index (12). In our cohort, the rate of disease progression in cases with *TP53* gene dysfunction was 75% (3/4). Among the 3 cases that progressed, 2 were classified as intermediate-risk SFTs. Taking our survival analysis results into consideration, we suggest that adding the parameter of *TP53* mutations and/or P53 immunophenotype could enhance the accuracy of identifying high-risk SFTs that may have been previously underestimated.

Similarly, SFTs with *TERT* promoter mutations were significantly associated with clinical features of malignancy, such as older age, larger tumor size, higher mitotic activity, necrosis, and higher grade (7, 13). In our series, mitotic activity was positively associated with *TERT* promoter mutations (p<0.001). We detected *TERT* promoter mutations in 7 intermediate-risk SFTs. Although not statistically significant, SFT progression rates with mutant-type *TERT* were higher than those with wild-type *TERT* in patients with intermediate-risk SFTs, consistent with previous observations by Machado et al. and others (14, 15). Thus, we suggest that *TERT* promoter mutations and *TP53* mutations may be used to identify patients at intermediate risk in the mDemicco system who may be more prone to developing disease progression.

Our data did not align with the previously reported clinical features of SFT. We found no significant differences in prognosis based on age, sex, primary site, tumor size, necrosis, tumor invasion, boundary, or P16 staining (refer to **Table 4**). It is important to consider the limitation of our results due to the relatively short follow-up period. In other studies, SFT recurrence or metastasis rates ranged from 10-40% occurring five years later (4), whereas in our series, the metastasis rate was only 7.4% during the available follow-up time (median time was 53 months, ranging from 1 to 136 months). Among the histologic parameters, only mitotic count exhibited a statistically significant correlation with both PFS and OS. Mitotic count is widely used in various risk models, such as the Demicco risk model, Pasquali model, Salas models (6), and G-score model (16), and has been identified as the most effective histologic parameter (14). However, considering the low mortality rate observed in our study, caution should be exercised when interpreting the results of survival analysis on overall survival. Therefore, we emphasize the need for longer follow-up studies to gain a more comprehensive understanding of patient outcomes in SFTs.

Additionally, it’s worth noting that we excluded all core biopsy samples, which might have resulted in inappropriately lower risk scores due to sampling bias. The discrepancy between biopsy and resection did not alter the risk score for patients with low-risk tumors (tumor size <10 cm and age <55 years). However, in larger tumors in older patients, low mitotic counts and the absence of necrosis on core biopsy should be cautiously interpreted due to the potential for underestimation (17).

Yasuhiro Ono (18) detected homozygous deletions of the *CDKN2/p16* gene in 7 of 27 (26.0%) patients with primary dural-based hemangiopericytoma (HPC). HPCs harboring *CDKN2/P16* deletion usually exhibit malignant behavior, but this genetic change was not associated with HPC progression. In our study, heterozygous deletions of *CDKN2A* were more common, suggesting potential differences in intracranial and extracranial SFT molecular genetics. However, long sample storage posed limitations in the interpretation of these results. Previous studies have indicated that high p16 expression is associated with malignancy and shorter disease-free survival time. *CDKN2A*, the gene encoding the cell cycle checkpoint protein P16, is considered a tumor suppressor gene and can be up-regulated or down-regulated in different tumors, with P16 also expressed in various other spindle cell tumors (19). The significance of the changes in *CDKN2/P16* remains unclear.

In conclusion, the mDemicco risk model showed limitations for prognosticating the risk of progress of SFTs in low-risk SFTs. Molecular factors such as *TP53* and *TERT* promoter mutations are correlated with poorer patient outcomes, providing valuable information for prognostic prediction of intermediate-risk SFTs stratified by the mDemicco risk model.

## Data availability statement

The original contributions presented in the study are included in the article/**Supplementary Material**. Further inquiries can be directed to the corresponding authors.

## Ethics statement

The studies involving humans were approved by Ethics Committee of The First Affiliated Hospital of Nanjing Medical University. The studies were conducted in accordance with the local legislation and institutional requirements. The human samples used in this study were acquired from Department of Pathology at the First Affiliated Hospital of Nanjing Medical University. Written informed consent for participation was not required from the participants or the participants’ legal guardians/next of kin in accordance with the national legislation and institutional requirements.

## Author contributions

CY: Conceptualization, Data curation, Formal Analysis, Investigation, Methodology, Visualization, Writing – original draft. JZ: Methodology, Writing – review & editing, Funding acquisition, Project administration, Resources. XL: Methodology, Writing – review & editing, Data curation, Formal Analysis. JY: Methodology, Writing – review & editing, Conceptualization. GC: Writing – review & editing, Data curation, Formal Analysis, Methodology. QF: Conceptualization, Methodology, Supervision, Writing – review & editing. QG: Conceptualization, Supervision, Writing – review & editing, Data curation, Funding acquisition, Methodology, Project administration, Resources. JW: Methodology, Visualization, Writing – review & editing.
