# Supplementary material for: Prognostic analysis of extrameningeal solitary fibrous tumor using the modified Demicco model: a clinicopathologic study of 111 Chinese cases
Source: Front Oncol. 2024 Jan 4;13:1272090. doi: 10.3389/fonc.2023.1272090 (PMC10796168; doi:10.3389/fonc.2023.1272090)
Supplement: Supplementary file 1 [file DataSheet_1.docx]

Supplement table 1

| Literature review | Points |
| --- | --- |
| Hassani et al. (9) | Confirmed the limitations of the mDemicco risk model in prognosticating aggressive tumor behavior |
| Yui chi yamda et al. (10) | Hypoglycemia and dedifferentiation were found to be independent prognostic factors of NAB2-STAT6positive/STAT6 IHC-positive SFTs for local recurrence, distant metastasis, and tumor death. |
| Thompson LDR et al. (11) | An orbital risk stratification for SFT is suggested, modifying current extrapleural schemes to account for a significantly higher local recurrence risk rather than metastatic disease while also taking into consideration the generally younger age at presentation and the smaller tumor size. |
